# PirVP genes causing AHPND identified in a new *Vibrio* species (*Vibrio punensis*) within the commensal *Orientalis* clade

**DOI:** 10.1038/s41598-018-30903-x

**Published:** 2018-08-30

**Authors:** Leda Restrepo, Bonny Bayot, Sebastián Arciniegas, Leandro Bajaña, Irma Betancourt, Fanny Panchana, Alejandro Reyes Muñoz

**Affiliations:** 1 Escuela Superior Politécnica del Litoral, ESPOL, Centro Nacional de Acuicultura e Investigaciones Marinas, CENAIM, Campus Gustavo Galindo Km 30.5 Vía Perimetral, P.O. Box 09-01-5863 Guayaquil, Ecuador; 2 Department of Biological Sciences, Universidad de los Andes, Bogotá, Colombia; 3 Max Planck Tandem Group in Computational Biology, Universidad de los Andes, Bogotá, Colombia; 4 Escuela Superior Politécnica del Litoral, ESPOL, Facultad de Ingeniería Marítima, Ciencias Biológicas, Oceánicas y Recursos Naturales, FIMCBOR, Campus Gustavo Galindo Km 30.5 Vía Perimetral, P.O. Box 09-01-5863 Guayaquil, Ecuador; 5 Department of Animal Health, Faculty of Veterinary Medicine and Animal Science, Universidad Nacional de Colombia, Bogotá, Colombia; 6 Center for Genome Sciences and Systems Biology, Department of Pathology and Immunology, Washington University in Saint Louis, Saint Louis, MO USA

## Abstract

Acute hepatopancreatic necrosis disease (AHPND) has extended rapidly, causing alarming shrimp mortalities. Initially, the only known causative agent was *Vibrio parahaemolyticus* carrying a plasmid coding for the mortal toxins *Pir*^*VP*^. Recently, it has been found that the plasmid and hence the disease, could be transferred among members of the *Harveyi* clade. The current study performs a genomic characterization of an isolate capable of developing AHPND in shrimp. Mortality studies and molecular and histopathological analyses showed the infection capacity of the strain. Multilocus sequence analysis placed the bacteria as a member of the *Orientalis* clade, well known for containing commensal and even probiotic bacteria used in the shrimp industry. Further whole genome comparative analyses, including *Vibrio* species from the *Orientalis* clade, and phylogenomic metrics (TETRA, ANI and DDH) showed that the isolate belongs to a previously unidentified species, now named *Vibrio punensis sp*. nov. strain BA55. Our findings show that the gene transfer capacity of *Vibrio* species goes beyond the clade classification, demonstrating a new pathogenic capacity to a previously known commensal clade. The presence of these genes in a different *Vibrio* clade may contribute to the knowledge of the *Vibrio* pathogenesis and has major implications for the spread of emerging diseases.

## Introduction

Shrimp production in Southeast Asia and Mexico had increased steadily in recent years, until the outbreak of a new disease, called acute hepatopancreatic necrosis disease (AHPND) or early mortality syndrome (EMS). This disease caused severe harm to the industry, resulting in significant economic loss to shrimp producers in China (2009), Vietnam (2010), Malaysia (2011), Thailand (2012), Mexico (2013) and Philippines (2015)^1,2^. The disease can affect both species of cultured penaeid shrimp, *Penaeus monodon* and *Penaeus* (*Litopenaeus*) *vannamei*. Mass mortality in cultured ponds has been observed 35 days after stocking, reaching up to 100 percent mortality within a few days after the first appearance of disease symptoms^3^. Clinical symptoms include lethargy, slow growth, empty stomach or midgut, and a pale to white atrophied hepatopancreas, with dead shrimp on the pond bottom^4^.

Members of the genus *Vibrio* comprise currently 14 recognized clades^5^, where the species are defined as clusters of strains with high phenotypic and genotypic similarities. Several *Vibrio* species are important causative agents of diseases in marine animals and humans. The pathogen identified by Tran *et al*.^6^ as the causing agent of AHPND correspond to a *Vibrio parahaemolyticus* strain. These authors reproduced the disease by infecting healthy shrimp with pathogenic bacteria. After verifying the Koch’s postulates, they concluded that AHPND has a bacterial etiology, identifying all isolates as members of the Vibrionaceae family and being closely related to *V*. *parahaemolyticus*. The genome of the pathogenic isolates of *V*. *parahaemolyticus* differs from non-pathogenic isolates because of the presence of two homologous genes related to the insecticidal toxin genes, *PirA* and *PirB* (*Pir*^*VP*^), described for the first time in *Photorhabdus sp*.^6^. The toxin coding genes are located on a plasmid (pV_AHPND_) and the plasmid has been identified as being present in all AHPND-causing bacteria^7^.

Until now, *V*. *parahaemolyticus* strains containing plasmids with the genes for putative virulence have been described in Thailand^8^, Mexico^9^, China^10^, Vietnam^8^ and South America^11^. Although there is genetic variability in the plasmid sequences, which has led to a description of a Mexican type, characterized by a 4,243 bp Tn3-like transposon insertion and a 9 bp small sequence repeat (SSR), both features being absent from the Asian type^8^. However, all plasmids contain the toxin genes^12^ and a group of transposase-coding sequence related with horizontal gene transference (HGT)^7^.

Sawabe *et al*.^13^ argued that the HGT within the *Vibrio* genus might occur between sister species, such as *V*. *cholerae* and *V*. *mimicus* (*Cholerae* clade) or *V*. *harveyi* and *V*. *campbellii* (*Harveyi* clade). To date, no evidence of HGT has been observed between species belonging to distantly related clades. Recently, the presence of the pV_AHPND_ has been discovered not only among *V*. *parahaemolyticus* strains, but also in other related species, in particular: *V*. *harveyi*, *V*. *owensii* and *V*. *campbellii*^6,14–16^, all species belonging to the *Harveyi* clade. For this reason, this clade is known to be the pathogenic clade for AHPND.

Most *Vibrio* species in the *Orientalis* clade are not causative agents of diseases, except *V*. *tubiashii* and *V*. *sinaloensis*, which have been associated with mortalities of bivalves and fishes^17,18^. In particular, there is no record of pathogens for crustaceans within this clade. The current study describes a new *Vibrio* species, *Vibrio punensis* *sp. nov.* strain BA55, belonging to the *Orientalis* clade and closely related to *V*. *orientalis* and *V. hepatarius*. Moreover, *V. punensis sp. nov*. strain BA55 carries the plasmid containing the toxin genes shown to be the cause of AHPND in shrimp. Our findings show that the gene acquisition capacity of *Vibrio* species goes beyond the clade boundaries that have been previously shown, demonstrating new infective capacity to a previously known commensal clade. The presence of these genes in a *Vibrio* clade not previously reported as pathogenic to crustaceans may contribute to the understanding of the *Vibrio* pathogenesis and has major implications for the spread of emerging diseases.

## Results

### *Pir*^*VP*^ gene detection, histopathologic analysis and typing using RAPDs

**Figure 1:**
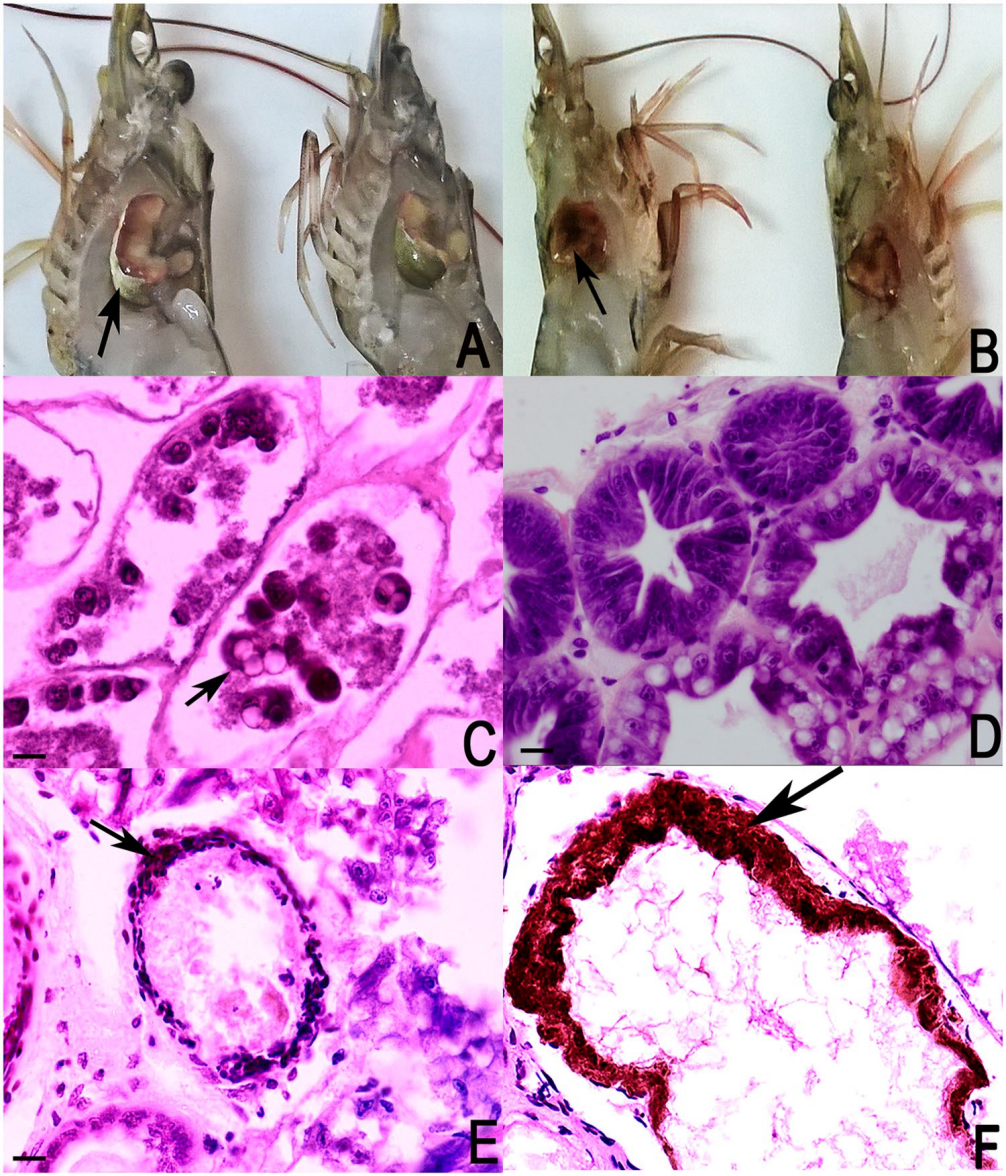
Photographs from healthy *P. vannamei* shrimp and shrimp infected with BA55 strain carrying *Pir*^*VP*^ genes associated with AHPND. (**A**) Left shrimp view. Gross signs of AHPND-infected shrimp (left): pale, atrophied hepatopancreas surrounded with a white membrane with smooth consistency (black arrow). Normal shrimp (right): normal size hepatopancreas with brownish color. (**B**) Right shrimp view. Gross signs of AHPND-infected shrimp (left): complete hepatopancreas destruction (black arrow). Normal shrimp (right): normal size hepatopancreas with brownish color. (**C**) Hematoxylin and eosin-stained histological sections of the hepatopancreas of *P. vannamei* from sick shrimps. AHPND pathology characterized by sloughing of hepatopancreatic tubule epithelial cells (black arrow). (**D**) Hematoxylin and eosin-stained histological sections of the normal shrimp hepatopancreas. (**E**) Histological sections show severe necrosis of the hepatopancreas tubules (black arrow), making it impossible to discriminate the different types of cells. (**F**) Histological sections show cellular detachment caused by a bacterial infection, evidencing the formation of melanized haemocytic nodules (black arrow).

Shrimp collected during a mortality event in 2015 in a South American farm showed the symptomatology of AHPND, with external signs of empty stomach, lethargy, discoloration of the hepatopancreas, with a white membrane of smooth consistency expanding through it (Fig. 1A,B). In the longitudinal sections of the shrimp, a whitish coloration was present in the hepatopancreas, indicating a degenerative disease in this organ (Fig. 1A).

Histopathologic evaluation showed severe necrosis of the hepatopancreas tubules, with detachment of the epithelial cells from the membrane to the lumen (Fig. 1C), making it impossible to discriminate between the distinct types of cells that are affecting the integrity of the tubules (Fig. 1E). A marked difference was observed when compared to hepatopancreas tubules of healthy shrimp, where it was possible to discriminate between embryonic and secretory cells, both appearing to be in good condition, without any detachment and maintaining the tubule structure without deformities (Fig. 1D). Severe hepatopancreas necrosis limited the observation of lipid cells due to the cellular detachment caused by a bacterial infection, evidencing the formation of melanized haemocytic nodules in the middle part of the hepatopancreas tubules (Fig. 1F).

**Figure 2:**
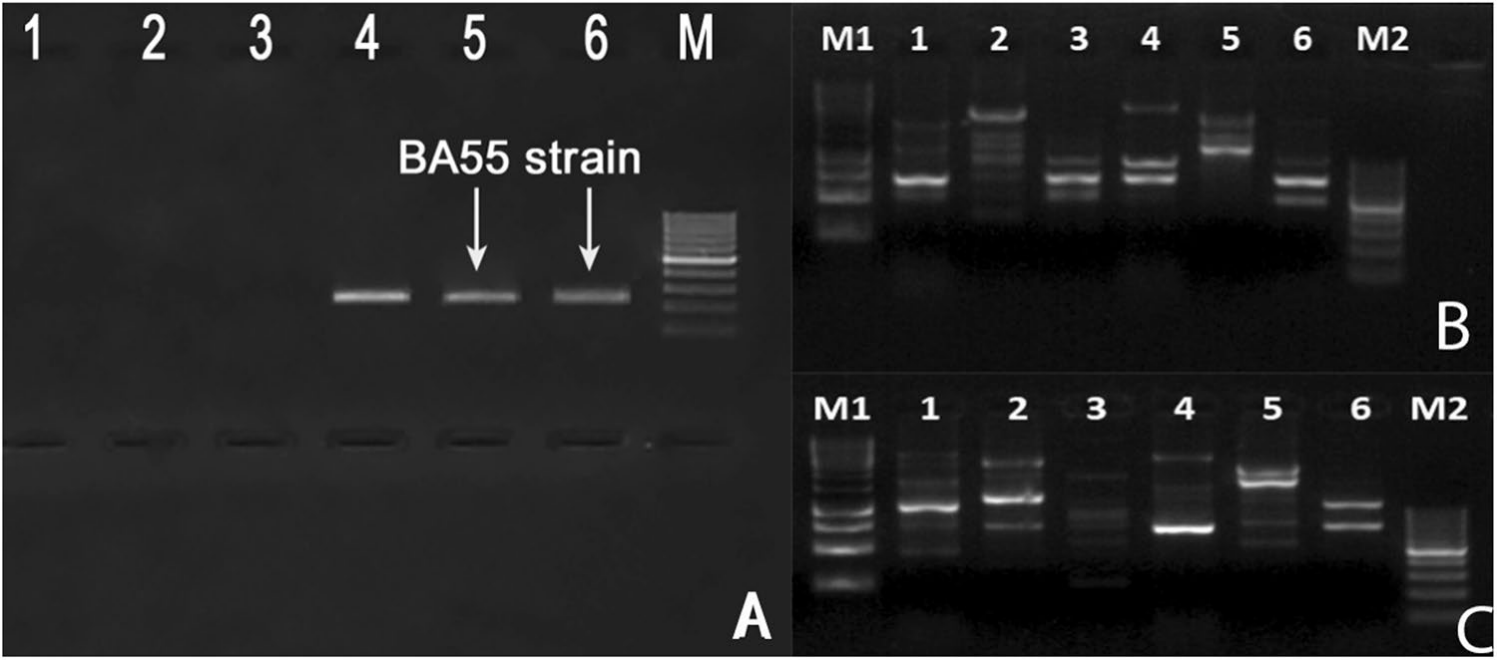
Identification of an isolate BA55 strain carrying *Pir*^*VP*^ genes associated with AHPND in *P. vannamei*. (**A**) Detection of *Pir*^*VP*^ genes. Lane 1: *V. parahaemolyticus* not containing the *Pir*^*VP*^ genes; lane 2: *V. harveyi* not containing the *Pir*^*VP*^ genes and lane 3: non-template control; lane 4: *V. parahaemolyticus* containing the *Pir*^*VP*^ genes; lane 5 and 6: PCR results from two independent DNA extractions from strain BA55; lane M: DL2000 DNA marker. (**B**) UBC101 amplicon profiles from the different isolates, lane 1: *V. parahaemolyticus*; lane 2: *V. harveyi*; lane 3: *V. campbellii*; lane 4: *V. orientalis*; lane 5: *V. hepatarius*; lane 6: BA55 strain. UBC 457 amplicon profiles from the different isolates, lane 1: *V. parahaemolyticus*; lane 2: *V. harveyi*; lane 3: *V. campbellii*; lane 4:*V. orientalis*; lane 5: *V. hepatarius*; lane 6: BA55 strain; lane M1: DL 10000 DNA marker; lane M and M2: DL1000 DNA marker.

Bacterial recovery from infected animals was dominated by a single morphotype, named strain BA55, which was confirmed to contain the *Pir*^*VP*^ genes associated with AHPND (Fig. 2A). RAPD (randomly amplified polymorphic DNA), a simple and cost-efficient assay to evaluate genetic variability, was used to discriminate between related *Vibrio* species. A unique pattern for the identified BA55 strain was observed when compared to different isolates of *Vibrio spp*. previously characterized at CENAIM (Fig. 2B,C), suggesting that the disease was caused by a strain with unique molecular characteristics different to previously isolated bacteria from the *Harveyi* clade. Furthermore, full 16S Sanger sequencing confirmed that the isolated strain did not belonged to the *Harveyi* clade (Fig. S1).

### Bacterial bioassay shows BA55 strain causes AHPND in shrimp

**Figure 3:**
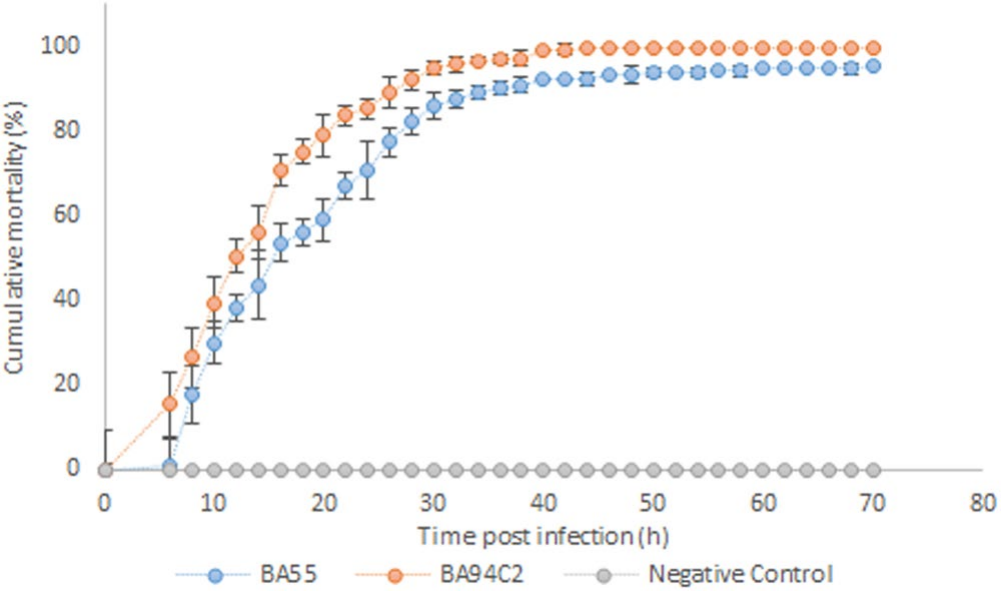
Shrimp cumulative mortality after being challenged with BA55 and *Vibrio parahaemolyticus* BA94C2 strains. Negative control was TSB 2% NaCl. Bars indicate standard deviations.

**Figure 4:**
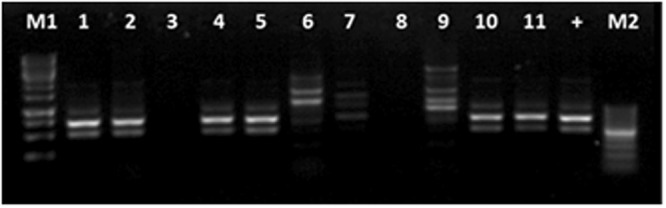
DNA banding pattern of bacterial isolates obtained from challenged shrimps infected with the pathogenic BA55 and *V. parahaemolyticus* BA94C2 strains. From challenged shrimp infected with the BA55 strain; Shrimp 1, lane 1: Large size and convex isolated (LCI); lane 2: Small size and convex isolated (SCI); lane 3: Large and plain isolated (LPI). Shrimp 2, lane 4: LCI; lane 5: SCI; lane 6: LPI. From challenged shrimp infected with the *V. parahaemolyticus* BA94C2 strain; Shrimp 3, lane 7: Large size isolated (LI); lane 8: Medium size isolated (MI); lane 9: Small size isolated (SI). Inoculum controls from BA55 strain, lane 10 and lane 11. Positive control, lane +. Lane M1: DL 10000 DNA marker; lane M2: DL1000 DNA marker.

The BA55 strain was used *in vivo* to challenge susceptible shrimp in triplicates starting at 2 × 10^6^ CFU ml^−1^. Shrimp mortality was observed after 10 h post infection, whereas the *V. parahaemolyticus* BA94C2 strain, at the same concentration, caused mortality after 8 h (Fig. 3). No mortalities were reported for the negative controls. Shrimp cumulative mortalities at 70 h post infection in both infected treatments were high (BA55: 95.2 ± 0.8%; BA94C2: 99.8 ± 0.7%), with a significant difference between treatments (P = 0.005, Kruskal‐Wallis test). Moribund shrimp from the BA55 infected treatment exhibited the same symptoms as observed on diseased *P. vannamei* collected from the shrimp farm. Moribund shrimp from different replicates infected with the BA55 strain were used for isolating the predominant *Vibrio* strains and recovering them in pure culture. The isolated strains were then further tested with the RAPD assay. The DNA banding pattern in these colonies was the same as those obtained from the BA55 strain isolated from the diseased cultured shrimp (Fig. 4) and used as an inoculum, thus confirming Koch’s postulates and confirming that the BA55 strain is a causative agent of AHPND. Additionally, the *PirA* and *PirB* toxin genes were detected by PCR in the isolated colonies recovered from moribund shrimp during the challenge, whereas the macerates of the negative controls remained negative (Fig. S2).

### *De novo* assembly of the BA55 strain genome sequence

Total DNA extracted from the isolated strain was used for *de novo* genome sequencing and assembled using 19,434,590 Illumina PE reads. A total of 75 contigs were obtained. Annotation and comparison to reference *Vibrio spp*. genomes showed evidence of two chromosomes (Chr 1: 3.2 Mbp long and Chr 2: 1.5 Mbp long) and a plasmid (pV_AHPND_; ~69 Kbp long) containing the toxin *Pir*, with two subunits: *PirA* and *PirB*. The average coverage was 46.3×, G + C content was 44.5%, 4,494 genes were predicted, and the total genome size was 4.7 Mbp (Table 1). An initial evaluation using BLAST among the predicted genes and a set of 76 other reference *Vibrio* genomes available in public databases (Table S1) showed no significant difference (less than 1% in the percent identity) among the scores obtained from the different *Vibrio* species, suggesting that this strain is equally distant from the previously known *Vibrio* species, potentially constituting an undescribed species.

**Table 1:** Genome characteristics of the strain BA55 draft genome.

Attributes	Values
Assembly size (bp)	4,727,353
Total number of contigs	75
Contig N50 (bp)	206,237
L50	10
GC content %	44.5
Number of CDS	4,494
Number of rRNAs	20
Number of tRNAs	142

### Phylogenetic analysis of the BA55 strain

The Maximum Parsimony (MP), Maximum Likelihood (ML) and Neighbor Joining (NJ) analysis of 16S rDNA showed a high identity between all *Vibrio* sequences analyzed and low support for the branches in all the inference methods used, suggesting a low resolution to resolve intra and inter-specific phylogenetic relationships within the *Vibrio* genus. We found that the closest neighbor of the strain BA55 was *V*. *brasiliensis* strain LMG 20546 (75% bootstrap values; Fig. S3) from the *Orientalis* clade. Importantly, although the 16S rDNA gene is commonly used for phylogenetic analyses, the sequence identities among *Vibrio* species tend to be very high making this gene an unsuitable marker for phylogenetic analysis within the genus, further confirmed by the low branching support and the incongruence on the clustering with the traditional *Vibrio* clades.

**Figure 5:**
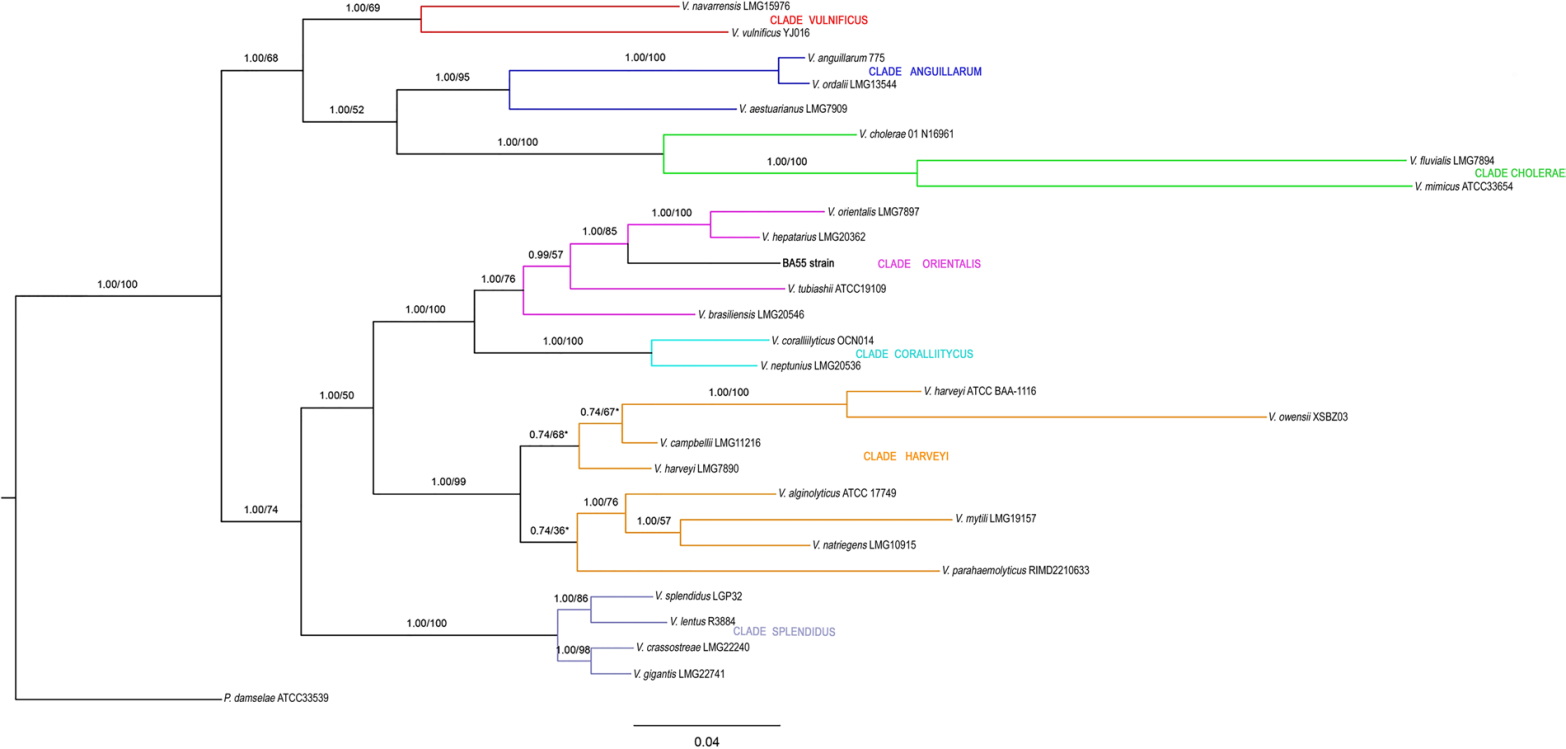
Phylogenetic concatenated reconstruction based on ftsZ, gapA, gyrB, mreB, topA, and 16S rRNA genes. A Bayesian based phylogeny on a concatenated multiple sequence alignment using the TIM + G evolutionary model is shown. Numbers above the branches represent the posterior probabilities and percent bootstrap values (one thousand bootstrap replicates). Reference sequences used were described by Sawabe *et al*.^5^ and sequences extracted from complete genomes. An independent Maximum Parsimony and Maximum Likelihood phylogenetic analyses were performed. Branches that were not conserved among the three topologies are shown with an asterisk. Scale bar represents number of substitutions per site. Color lines represent each *Vibrio* clade as follows: *Vulnificus* clade in red; *Anguillarum* clade in blue; *Cholerae* clade in green; *Orientalis* clade in purple; *Coralliilyticus* clade in light blue; *Harveyi* clade in orange; *Splendidus* clade in gray. Our results show evidence that the strain BA55 is related to *V*. *hepatarius* and *V*. *orientalis*. The outgroup used was *Photobacterium damselae*.

In order to gain a higher phylogenetic resolution, the phylogenetic trees derived from the concatenated nucleotide sequences of six commonly used MLST genes (ftsZ, gapA, gyrB, mreB, topA, and 16S rRNA genes) showed that the strain BA55 branched deep within the *Orientalis* clade, indicating a sister group relationship with *V*. *orientalis* and *V*. *hepatarius*. In fact, these three species form a monophyletic group within the *Orientalis* clade, distant from other known AHPND-causing bacteria and all the *Vibrio* within the *Harveyi* clade (Fig. 5). In addition, MP, ML, NJ and Bayes analyses revealed consistent phylogenetic tree topologies. The BA55 strain was phylogenetically distant from the *Anguillarum* clade (89.01% ± 3%), *Vulnificus* clade (87.69% ± 1%) and *Cholerae* clade (81.46% ± 3%), and more closely related to members of the *Orientalis* clade: *V. hepatarius* (96.32%), *V. orientalis* (95.57%) and *V*. *tubiashii* (89.85%). The closest clades to the BA55 strain were the *Coralliilyticus* and the *Harveyi* clades (90.12% ± 2% and 89.23% ± 4%), the latter being the pathogenic clade for AHPND, containing *V*. *parahaemolyticus*, *V. harveyi*, *V. owensii* and *V*. *campbellii*, the species where the presence of the pV_AHPND_ plasmid has been previously reported^6,14–16^. Percentages shown are the average sequence similarity, calculated with the proportion of nucleotide sites that differ between sequences and divided by the total number of sites for the comparison; in our case, based on the six protein-coding housekeeping genes of the BA55 strain.

Also, we show the phylogenetic reconstruction supporting the taxonomic position of the strain BA55, well-differentiated within the *Orientalis* clade (Fig. S4). Phylogenetic trees based on the concatenated partial sequences of the five housekeeping genes reconstructed by the NJ, ML and Bayes methods supported the independent and robust position of the strain BA55 within the *Orientalis* clade, especially the closest position relatives to *V. orientalis* and *V. hepatarius* and maintaining a low percentage of similarity with *V. tubiashii*, *V. bivalvicida* and *V. europeaus*. *V. crossai* maintains its position with *V. brasiliensis*.

For calibration of the evolutionary rates and estimation of the divergence times, we used a set of representative genomes from the *Vibrio* genus and analyzed the rate of amino acid substitutions in the six housekeeping genes. The lowest radiation time value for our strain BA55 was obtained with *V*. *hepatarius* (Table 2), revealing that these species are closely related. The different phylogenetic trees reconstructed from species of the *Harveyi* and *Orientalis* clades showed a consistent branch pattern, in particular for the relative position of the BA55 strain within *Orientalis* clade (Fig. 5).

**Table 2:** Clades and subclades used for MLST analysis.

Clade	Species included	No. of species	GC (mol%)^a^	MLST (%)^b^	AAI (%)^c^	Phi test (P value)^d^	Habitat^e^
*Anguillarum*	*V. anguillarum*, *V. aestuarianus and V. ordalii *	3	43–46	95.5	99	87.0–98.3 95.8–99.8	Brackish water, seawater and fish
*Cholerae*	*V. cholerae*, *V. mimicus, V. furnissii, V. fluvialis, V. metschnikovii and V. cincinnatiensis*	6	44–50	87	96.3	83.4–94.4 93.5–99.8	Brackish water, seawater
*Coralliilyticus*	*V. coralliilyticus* and *V. neptunis*	2	45–46	97	99.7	94.2 99.5	Seawater, bivalves and rotifers
*Harveyi*	*V. harveyi*, V. *campbellii*, *V. parahaemolyticus*, *V. alginolyticus*, *V. mytili*, *V. natriegens*, and *V. rotiferianus*	7	42–48	91.3	97.8	86.7–96.0 96.9–99.9	Seawater, salt marsh mud and marine animals
*Orientalis*	*V. orientalis*, *V. hepatarius*, *V. brasiliensis*, *V. sinaloensis*, *V. tubiashii* and BA55 strain	6	43–46	95.9	99.2	100	Brackish water and seawater
*Splendidus*	*V. splendidus*, *V. crassostrea*, *V. cyclitrophicus*, *V. chagasii*, *V. fortis*, *V. kanaloaei*, *V. lentus*, *V. gigantis*, *V. pelagius*, *V. tasmaniensis* and *V. pomeroyi*	11	39–47	98.2	99.8	88.8–93.7 97.1–98.9	Seawater and marine animals
*Vulnificus*	*V. vulnificus* and *V. navarrensis*	2	45–48	93.6	98.8		Sewage, seawater and oyster
*Damselae*	*P. damselae*	1	42				Seawater and fish

^a^GC content c*alculated based on six genes*.

^b^MLST *percentage of concatenate similarity between sequences*.

^c^AAI *Raw average amino acid identity (10)*.

^d^Phi test. The phi test was conducted for clades that included at least four species.

^e^Habitat described for Sawabe *et al*.^13^.

### Sequence analysis of pV_AHPND_ in the *strain BA55*

**Figure 6:**
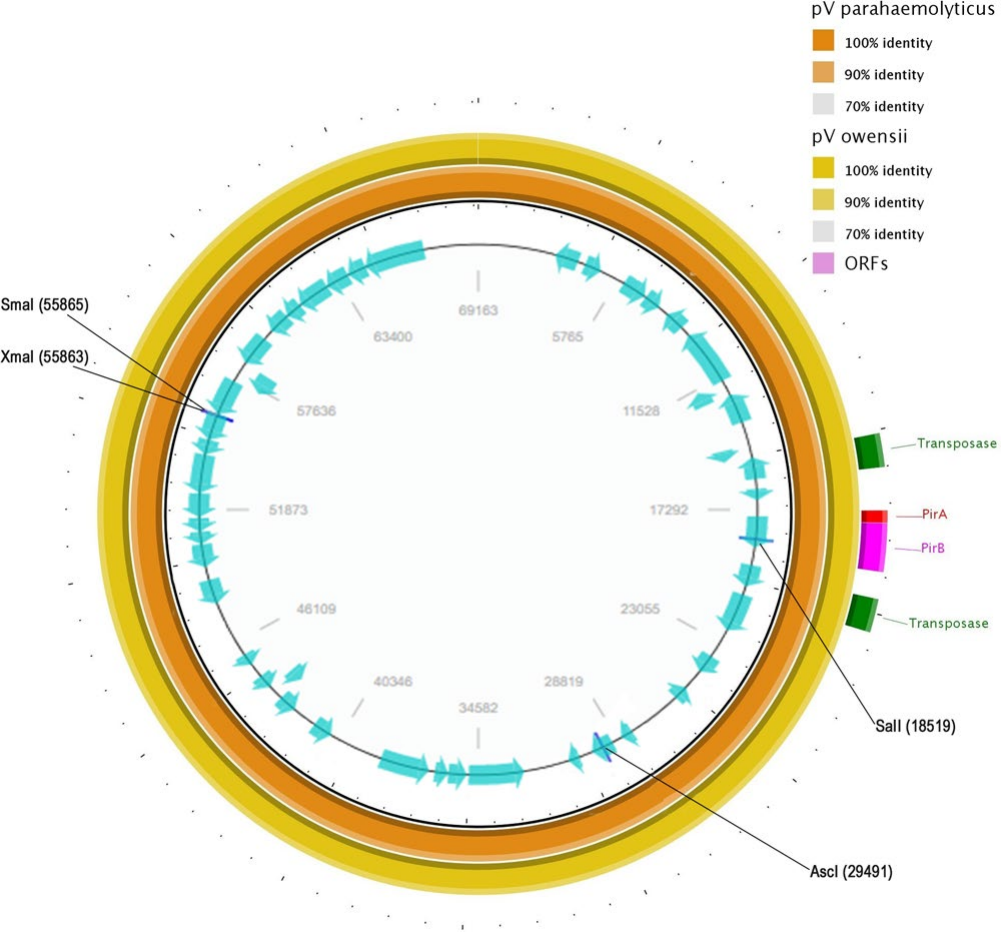
Sequence map of the AHPND-associated plasmid pV_AHPND_ in strain BA55. First two outer circles represent the BLAST comparison with the plasmid of *V. parahaemolyticus* (orange) and *V. owensii* (yellow). The internal ring represents the predicted open reading frames (ORFs) shown as arrows in blue, direction of arrowhead represents the transcriptional orientation. The transposase genes in green, red and purple represent the *PirA* and *PirB* genes correspondingly.

The plasmid pV_AHPND_ found in the strain BA55 was assembled in a single 69,163 bp contig, with a mean GC content of 44.9% and 53 putative ORFs. A homologue of the insecticidal *Photorhabdus* insect-related binary toxin *PirA* and *PirB* was also identified in this plasmid. The toxin genes are flanked by an arrangement of SWAT-3 type transposases (V_SWAT3_ like transposases), suggesting a potential mechanism of HGT between different *Vibrio* populations^11^. Importantly, no homologous sequences for the transposases were identified in any of the genomic contigs and evidence for a robust assembly of the plasmid was supported by pair-end reads agreement and an average coverage of 1,200X throughout the plasmid sequence. Xiao *et al*.^19^ reported that similar Tn903-like transposons were detected in either the plasmid (*V. owensii*, *V. harveyi* and *V. parahaemolyticus)* or within the chromosomal genome (*V. anguillarum* and *V. campbellii)* of species belonging to the *Harveyi* clade, but they have not been reported in the *Orientalis* clade. The comparison of the plasmid sequences from the *V. parahaemolyticus* (accession number: KM067908), *V. owensii* (accession number: KX268305), both from the *Harveyi* clade, and the strain BA55 showed a high percentage of similarity (99.97 average percent identity) demonstrating that they are closely related to each other (Fig. 6). It is important to note that no similar plasmid has been described within species from the *Orientalis* clade.

### Comparative genome analysis

**Figure 7:**
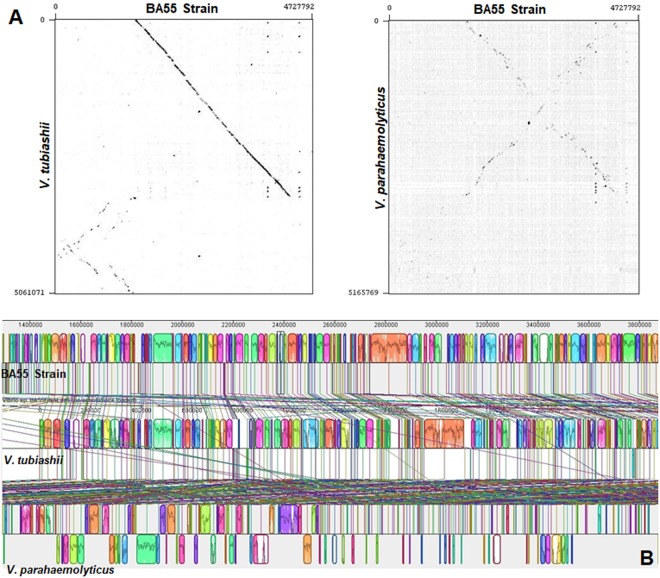
Whole genome alignments between *V. punensis* strain BA55, *V. tubiashii* strain ATCC 19109 and *V. parahaemolyticus* RIMD 2210633. (**A**) Dot-plots of nucleotide identities of the BA55 strain against *V. tubiashii* strain ATCC 19109 (left) and BA55 strain against *V. parahaemolyticus* RIMD 2210633 (right). (**B**) Nucleotide-based multiple genome alignment of the same genomes. Homologous blocks are shown as identically colored regions and linked across the genomes. Rearrangements are shown as homologous regions in different genomic locations.

**Figure 8:**
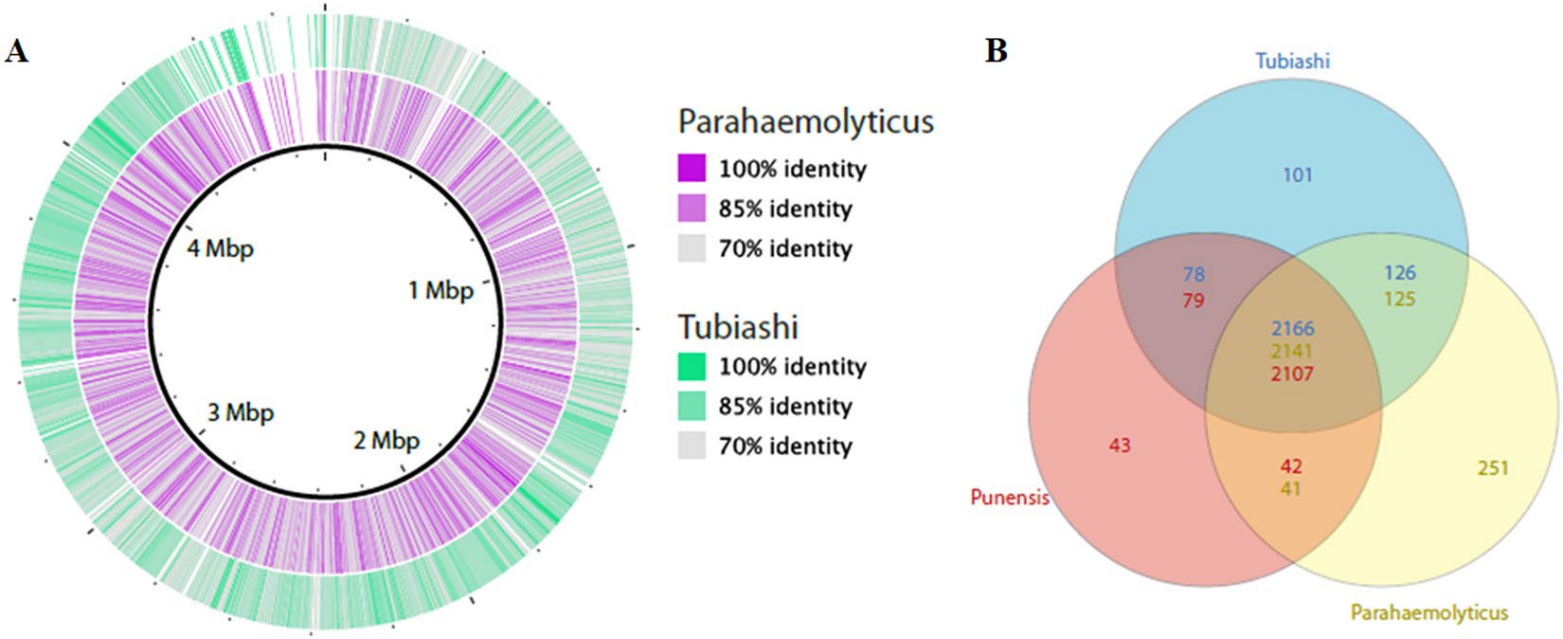
Comparative analysis of BA55 strain with *Vibrio tubiashii* ATCC 19109 and *Vibrio parahaemolyticus* RIMD 2210633. (**A**) Circles 1 and 2 show the BLAST comparison with *V. tubiashii* (green) and *V. parahaemolyticus* (purple). (**B**) Venn diagram showing unique and shared genes based on the RAST subsystems annotation among the three *Vibrio* species used before (BA55 strain, *V. tubiashii* and *V. parahaemolyticus*). Intersects show different number of genes for each genome due to potential paralogs within the genomes.

The comparative genomic analyses supported the hypothesis of the strain BA55 as a member of the *Orientalis* clade. The evidence came from multiple genome alignments and the evaluation of genomic architectures using Blast. The pairwise genome alignment between the genomes of the strain BA55 with *V. tubiashii* (*Orientalis* clade) and *V. parahaemolyticus* (*Harveyi* clade) suggested a closer relationship to *V. tubiashii* than to *V. parahaemolyticus* (Fig. 7A) as there was a higher number of rearrangements with the *V. parahaemolyticus* strain (Fig. 7B). The closer similarity was observed both when comparing the genomes at the nucleotide and amino acid level. Blast analysis showed a higher average percent identity with *V. tubiashii* (91%) compared to *V. parahaemolyticus* (88*%)* (Fig. 8A). At the amino acid level, the shared genes with a RAST annotated function between *V. parahaemolyticus* and the strain BA55 contained mainly essential genes that are needed for growth or viability (that were also shared with *V. tubiashii* and are likely part of a *Vibrio* core genome) and only 42 genes uniquely shared with *V. parahaemolyticus*, whereas *V. tubiashii* and the strain BA55 shared genes that are necessary for adaptation, including the genes for growth in different environments, which are associated with biofilm formation and bacterial communication like the Acetyltransferase SypM (Fig. 8B inset). This suggests the existence of gene clusters unique to the *Orientalis* strains distinct from the other *Vibrio*’s core genes. Thirty-two genomic islands (GIs) were detected for the strain BA55 (Table S2) including sequences coding for mobile genetic elements, as prophages and transposons, and several recombination-related proteins as integrases, recombinases and transposases. The gene comparison represented by the Venn diagram showed different number of shared genes between pair of genomes, which is due to homologs within the same genome (paralogs) (Fig. 8B inset).

### *Pir*^*VP*^ gene causing acute hepatopancreatic necrosis disease identified in a novel species from the *Orientalis* clade

**Figure 9:**
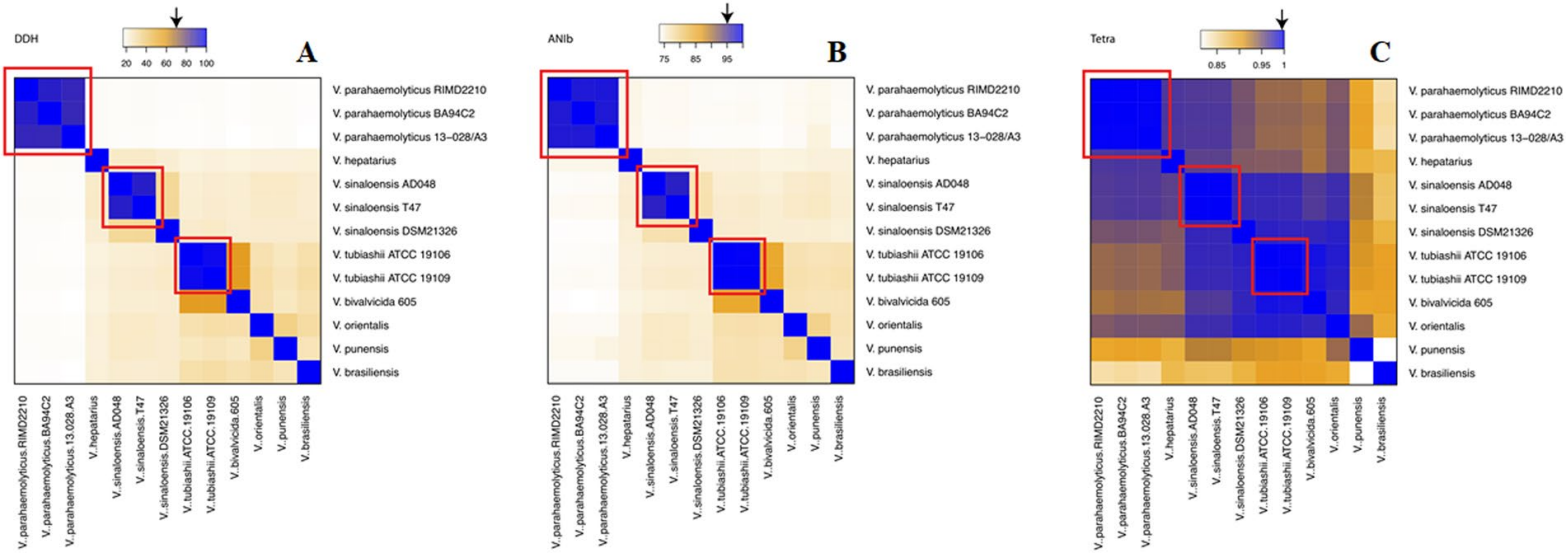
Heatmaps representing metrics for the evaluation of species circumscription among *Vibrio* strains. The extent of nucleotide identity was calculated by different indices for species circumscription: DDH (**A**), ANIb (**B**) and Tetra (**C**) as illustrated. The key color was constituted by orange and blue as indicated. Arrows shows the reported cut-off for species a boundary. Red boxes shows clusters of genomes within the reported cut-off.The species and strain number of samples are shown.

In order to calculate the distances among the genomes on seven members of the *Orientalis* clade (*V. hepatarius*, *V. sinaloensis*, *V. tubiashii*, *V. bivalvicida*, *V. orientalis*, *V. brasiliensis* and BA55 strain) and one member of the *Harveyi* clade (*V. parahaemolyticus*) we used the Genome-to-Genome Distance Calculator (GGDC 2.1)^20^. The first evaluated method was the *in silico* DDH (DNA-DNA hybridization) analysis. Our results show that the BA55 strain displayed DDH values below 70% with every other strain analyzed. This cut-off has been traditionally used for the delineation of species in prokaryotes, for both *in silico*^21^ and experimental analysis^22^ (Fig. 9A). For all pairwise comparisons made, the highest value obtained was 36% with *V. orientalis* (Table S3). In addition, the percentage of differences between G + C content was >1%, corresponding to values obtained traditionally between distinct species^20^.

ANI calculations were performed using MUMmer (ANIm) and BLAST (ANIb) algorithms^23^. Previous studies using ANIm and ANIb for species classification in the *Orientalis* clade had suggested a species boundary of 95–96%^20^. Our results are consistent with those observations given, that all known strains within a given species showed pairwise ANIm values above the threshold, except for *V. sinaloensis* strain DSM21326, concordant to the DDH results (Fig. 9B, Table S3) furthermore, the ANIb results confirm that BA55 is a species distinct from others available, with an ANIb value of <79% and an ANIm of <86% (Fig. 9B, Table S3). The discrepancy between the ANIb and ANIm values obtained is consistent with previous reports from Li *et al*.^24^ and Dubert *et al*.^25^ where they demonstrated the lack of agreement between ANIb and ANIm when the compared genomes show low correlation values (ANIb < 75% and <77%, respectively), in our case the ANIb value is <79%.

Furthermore, it has been argued that the oligonucleotide frequencies carry a species-specific signal^26^. For this reason, statistical calculation of oligonucleotide frequencies (TETRA) from pairwise comparisons was performed. TETRA values indicated that the strain BA55 is more related to *V. orientalis* strain CIP102891 (Fig. 9C). When comparing with the DDH and ANI cut-offs for species demarcation, TETRA values between relative species were above 99.5%, supporting their affiliation to the same species and between the *Orientalis* clade, this value can be proposed as a plausible cut-off. In contrast, the highest values for the BA55 strain were 93% for the comparison with *V. orientalis* and 92.4% with *V. sinaloensis* (Table S3). Together with the results based on *in silico* DDH and ANI we also suggest that *V. sinaloensis* strain DSM21326 should be reclassified, as it does not comply with the species described cut-offs as the other strains of *V. sinaloensis* does.

Based on all previously reported evidence and the nucleotide composition values, we proposed the strain BA55 as a new specie named *Vibrio punensis sp*. *nov.* strain BA55, belonging to the *Orientalis* clade. This novel species carries the plasmid pV_AHPND_ giving it the pathogenic characteristics that had been only previously reported on the *Harveyi* clade.

## Discussion

The visible symptoms observed in dying shrimp collected during the challenge test were similar to those observed in diseased shrimp collected from farms. The results of our challenge test indicated that the isolated BA55 strain triggered a high mortality, although lower than the strain BA94C2, and similar to other AHPND strains reported by Tran *et al*.^6^ and Nunan *et al*.^4^, where shrimp reached 100% mortality at 48 hours post infection trough immersion assays in experimental tanks, at an approximate bacterial density of 2 × 10^6^ CFU ml ^−1^. The results obtained from the challenge test using the BA55 strain compared with the control, and the fact that it contains the *PirA* and *PirB* genes suggest that the toxin, as is the case in other AHPND-causing *Vibrios*, is responsible for the mortality of the infected shrimp.

Current microbial taxonomy is being revolutionized by the generation of full-length microbial genomes from uncultured microorganisms and full genome assembly from metagenomic projects^27^ requiring a full set of new taxonomical tools and standards. To date, DNA-DNA hybridization (DDH) has been the “gold standard” for bacterial species demarcation, as it provides a numerical threshold for a species boundary^28^. Nevertheless, some authors suggest the necessity of a new approach due to the labor-intensive and error-prone nature of DDH experiments^20–28^. According to Richter and Rosselló-Móra *et al*.^23^ the scientific community needs methods that offer a similar resolution of DDH and simultaneously allow the construction of databases to retrieval of any information for comparative purposes. Therefore, the average nucleotide identity (ANI) method was developed and it seems to be the best alternative for a gold standard method^29^. Richter and Rosselló-Móra^23^; Ramasamy *et al*.^27^ and Meier-Kolthoff *et al*.^20^ among others, have made efforts to validate ANI as a mirror for DDH and that in conjunction with TETRA values, has been proposed as a plausible substitute for DDH, although further testing should be performed in particular clades. Given the mounting evidence, the recent use of such metrics for proposing other *Vibrio* clades and species^13–15^, and the results shown in our study, we propose the BA55 strain as a novel species, named *Vibrio punensis* sp. BA55.

Researchers have proposed that HGT drives evolution by incorporating genetic material from one organism to another. In this specific case, it is plausible that through HGT, the *Vibrio punensis sp*. *nov.* strain BA55 had become pathogenic. This shows that the gene transfer and the capacity of this new species to use these foreign genes is offering a new infective capacity to a species and a clade that has not previously been reported as a pathogen in crustaceans. Previously, it was only reported that species of the *Harveyi* clade present the ability to use *Pir*^*VP*^ genes, carried in plasmids, to cause AHPND. The current phylogenetic study revealed that these genes could be found in new species and thus enhance their infective capacity, thereby potentially increasing the complexity of causative agents of AHPND and aggravating the threat to the cultured shrimp industry.

Current developments in sequencing technologies and microbial ecology are changing the perception that we had about commensals and pathogens and blurring the lines separating them. Common aquatic environments are composed of thousands of different bacterial species displaying complex interactions and where selective pressures award those capable of gaining new properties that enhance the fitness of the surviving species. The *Vibrio* genus is a large and complex taxon that includes free-living bacteria found in aquatic environments but with the potential of residing in different animal hosts. The specific interactions with the host ranges from beneficial, as those described in *V. hepatarius* used as probiotic in shrimp^30^ to pathogenic, such as *V. cholerae*, *V. parahaemolyticus*, *V. vulnificus* and *V. alginolyticus*^31–33^. Once related species with different adaptation strategies to an environment collocate within a confined space under strong selection (such as different *Vibrio* species within the gut of a crustacean animal), it constitutes the perfect setup for the occurrence of HGT. This scenario could be the cause for a previously commensal bacterium to become pathogenic. The same principle applies to any other environment where commensal or symbiotic microorganisms potentially interact with pathogens, thus, increasing the chances of horizontal gene transfer of such virulence markers, which not necessarily limited to toxins, but also to potential antibiotic resistance in environments such as the human gut.

## Materials and Methods

### Isolation of bacterial strains

On July 2015, stomachs and hepatopancreas from AHPND positive shrimp collected during a mortality event in 2015 in a South American farm were removed and separately macerated to obtain mixed cultures. These cultures were subdued to subculture on TCBS (thiosulfate citrate bile salts sucrose) agar plates to obtain individual colonies and were further purified on TSA (tryptic soy agar) plates and pure isolates were then cryopreserved at −80 °C. A total of 39 bacterial strains were isolated from *P. vannamei* juvenile shrimp. Bacterial strains were characterized using morphological and biochemical criteria following the schemes of Alsina & Blanch^34^ and bacterial typing were conducted by 16S rRNA sequencing. Amongst those, a predominant bacterial strain coded as BA55 was selected for further analysis.

### *Pir*^*VP*^ gene detection

Bacterial samples were centrifuged at 4,000 g for 10 minutes and decanted. The resulting pellet was resuspended in 500 µl extraction STE buffer (10 mM Tris-HCl, 1 mM EDTA and 100 mM NaCl, and 0.2 mg/ml proteinase K; Invitrogen, Carlsbad, CA) and incubated at 55 °C for 2 hours. After cell lysis, purification was carried out with phenol-chloroform and precipitation with ethanol, following procedures by Maniatis *et al*.^31^ with minor modifications. Each supernatant was removed and extraction with an equal volume of phenol-chloroform-isoamyl alcohol (25:24:1) was performed, followed by extraction with chloroform-isoamyl alcohol (24:1). DNA was recovered by the addition of 1 volume of isopropanol followed by centrifugation at 10,000 g for 10 min. The DNA pellet was washed with 70% ethanol, dried, and resuspended in 50 µl of ultrapure water pH 7.0. DNA extracts were stored at −20 °C for further analysis. DNA concentrations were determined using a NanoDrop 8000 Spectrophotometer (NanoDrop 2000, Thermo Fisher Scientific Inc., Wilmington, USA).

The presence of the main genes associated to AHPND (*Pir*^*VP*^) were tested on all 39 isolated bacterial strains using a nested PCR method, with primers previously described, to obtain amplified products of 1,269 and 230 bp (Table S4). Likewise, *PirA* and *PirB* genes were amplified separately with specific primers aimed at generating PCR products of 284 and 392 bp for *PirA* and *PirB*, respectively (Table S4^12^).

### RAPD analysis

Two primers (UBC101, 5′-GCGCCTGGAG-3′and UBC 457, 5′- CGACGCCCTG -3′) were selected from an initial screening with 10 decanucleotide primers, for the evaluation of RAPD pattern bands of the bacterial strains: BA55, AHPND *V. parahaemolyticus* BA94C2^11^ and pathogenic non-AHPND strains (*V. parahaemolyticus, V. harveyi, V. campbellii*, acquired from the Microbiology Laboratory, Universidad Santiago de Compostela and *V. hepatarius*, acquired from the Microbiology Laboratory, CENAIM-ESPOL). All strains were grown overnight at 28 °C on TSA. One colony of each strain was suspended in 200 µl STE buffer and the DNA extraction was performed as described above. PCR was carried out in a 10 ml reaction volume containing 10 mM Tris-HCl, pH 8.3, 50 mM KCl, 3 mM MgCl2, 100 mM of each dNTP, 0.4 mM of the arbitrary primer, 2 µl of genomic DNA and 1 Taq DNA polymerase (Invitrogen). Amplifications were performed using a Thermo Fisher thermoblock under the following conditions: 4 min initial denaturation at 94 °C, followed by 39 cycles of 5 sec denaturation at 94 °C, 45 sec annealing at 46 °C, and 1 min elongation at 72 °C, with a final elongation step at 72 °C for 5 min. The PCR products (5 μl) were analyzed on a 1.5% agarose gel stained with SYBR Safe DNA Gel Stain (Thermo Fisher), which was then visualized under UV transillumination and photographed using an E-Gel Imager (Thermo Fisher).

### Immersion challenge test

The pathogenicity of the BA55 strain was evaluated by an immersion challenge test as described by Tran *et al*.^6^, with minor modifications. Briefly, a single colony of the BA55 strain was picked and re-suspended in 30 ml of sterile TSB (tryptic soy broth culture medium with 2% NaCl). The culture was incubated for 18 h at 28 °C on a rotary shaker (200 rpm) and the bacterial density was determined by a microplate luminometer (Varioskan™, Thermo Fisher Scientific). One ml of this solution was transferred to 50 ml of TSB medium and cultured as described above for 10 h. The suspension was plated onto TSA after serial dilutions to determine the colony-forming units of the isolate (CFU ml^−1^). The *V. parahaemolyticus* BA94C2^11^ strain was used as positive control under the same conditions.

For the infection, each strain was cultured in TSB for 12 h at 28 °C, to reach 2 × 10^9^ CFU ml^−1^. Then, a 15 min immersion was performed on flasks containing 40 healthy shrimps (2.5 ± 0.5 g) per flask and a solution of bacterial suspension with saline water, to achieve a bacterial density of approximately 2 × 10^8^ CFU ml^−1^. Immersed animals were transferred into experimental aquariums filled with 40 L of filtered and UV sterilized seawater and continuously aerated. After transfer, a solution of bacterial suspension was immediately added directly to the experimental tanks containing clean seawater (filtered and UV sterilized), to reach an approximate bacterial density of 2 × 10^6^ CFU ml^−1^. A negative control (40 shrimp immersed in sterile TSB, without bacterial strains) was also included. All treatments, including the control, had 10 replicates. During the assay, shrimp were monitored for mortality every 2 h. Difference of cumulative mortality at 70 h post infection among infected treatments was analyzed with the non-parametric Kruskal-Wallis test by using the R software^35^.

### Re-isolation of bacteria

Moribund shrimp from infected treatments were collected 12 h post infection. Samples from the hepatopancreas and stomachs were removed and macerated in sterile saline solution. One hundred microliter of serial dilutions (10^−1^, 10^−2^ and 10^−3^) containing macerated hepatopancreas and stomachs were inoculated on TCBS agar and incubated at 28 °C for 18 h, and the three most predominant colonies were purified on TSA plates (Table S5). Following this, the DNA of the colonies was extracted and stored for subsequent comparisons with the tested isolates using RAPD assay^36^, and *Pir*^*VP*^ genes detection, as described above. Macerates of shrimp hepatopancreas and stomachs of negative controls were also analyzed for *Pir*^*VP*^ genes detection. Shrimps were analyzed individually.

### Histopathologic analysis

Shrimp samples were preserved with Davidson AFA fixative for histopathology analysis. Tissues were processed in accordance with the histological routine procedure^37^. Sections were cut at 5 μm and stained with Mayer-Bennet hematoxylin and eosin. Tissues were examined for histopathological changes.

### Genome sequencing and *de novo* assemble

The genome of the predominant bacteria strain, BA55, was sequenced using Illumina MiSeq PE150 platform. The library was constructed with Illumina Paired-End DNA Sample Kit (Illumina Cambridge Ltd, UK). Raw Illumina FASTQ files were demultiplexed and quality-filtered using FastQC^38^. Any reads containing ambiguous base calls or barcode errors were discarded. *De novo* assembly was performed using Spades^39^. The generated contigs were used as seeds into Contiguator^40^ to polish it, through an alignment of raw reads on contigs by BLAST^41^. The complete genome sequence was deposited at DDBJ/EMBL/GenBank under the accession PRJNA412371.

### Genome annotation

The genome of the BA55 strain was annotated using GLIMMER^42^ and NCBI Prokaryotic Genome Annotation Pipeline^43^. In addition, other 76 *Vibrio* genomes from the GenBank database (Table S1) were used for multilocus sequence typing (MLST) phylogeny. These 76 genomes were re-annotated by RAST^44^ and the RNA regions were characterized using RNAmmer^45^. Then, all the predicted ORFs were translated and compared by a homology search (BLAST) against non-redundant protein databases from NCBI (E-value threshold = 10^6^ and minimal alignment length ≥80%), Swiss-Prot^44^, Clusters of Orthologous Groups (COG^46^), Kyoto Encyclopedia of Genes and Genomes (KEGG^47^) and Gene Ontology (GO^48^). To evaluate the functional and evolutionary relationship between the 76 *Vibrio* genomes and the assembled genome of the BA55 strain, a comparison among those genomes was performed to evaluate sequence similarities.

### 16S rDNA and multilocus sequence typing phylogeny

An initial phylogenetic analysis was performed with a dataset of 862 16S rDNA sequences obtained from the NCBI Reference Sequence Database (RefSeq), together with the 16S rDNA sequences extracted from the 77 complete *Vibrio* genomes (including the genome of the BA55 strain). Before performing all the phylogenetic analyses, a dereplication of the sequences by species was performed, retaining only one representative sequence per specie, obtaining a final set of 216 sequences (Fig. S5). All non-redundant sequences were aligned with MEGA 6.0^49^ using the MUSCLE algorithm. Phylogenetic trees were constructed using Maximum Parsimony (MP), Maximum Likelihood (ML) and Neighbor Joining (NJ) methods. We use JModeltest 2.0^50^ to test the evolution models based on the hierarchical likelihood ratio test, determining that the TIM + G model best fit the data^51^. The nucleotide substitution model chosen was consistent with previous reported models of *Vibrio* evolution^13^. The evolutionary distances between the species of *Vibrios* were estimated with MR. Bayes, considering the branch-length and divergence time for model selection across a wide range of phylogenetic and evolutionary models^52^.

To establish a more robust inference of the evolutionary history of the *Vibrio* genus, the same phylogenetic analyses described before were repeated, using a MLST analysis, by evaluating partial sequences of six commonly used, phylogenetic marker genes: ftsZ, gapA, gyrB, mreB, topA, and 16S rRNA from *Vibrio* species (70 sequences) reported by Sawabe *et al*.^5^, complemented by the 77 genomes used for re-annotation (including the genome of the BA55 strain). GenBank accession numbers are listed in Tables S1 and S6. We used the TCS program^53^ to estimate a phylogenetic network estimation among all *Vibrio* species used for MLST analysis, to choose the 21 more related to the BA55 strain.

Recombination was evaluated with the Phi test using the concatenated sequences of *Vibrios*, reported by Sawabe *et al*.^13^ Recombination was detected in gyrB (*P* = 6.4 × 10^−3^), gapA (*P* = 6.8 × 10^−7^) and topA (*P* = 4.5 × 10^−2^) in agreement with the conflicting phylogenetic splits (Table 2). The analysis was performed with SplitsTree4^54^, a framework for building phylogenetic trees and networks. Detecting recombination is basically a statistical endeavor and ideally *in vitro* experimental work should be carried out in order to confirm the ability of *Vibrios* to carry out recombination in the loci analyzed^13^.

### Comparative genome analysis

MAUVE software^55^ was used to perform whole genome alignments and comparisons. The genome of the BA55 strain was aligned to the two most cured, well-annotated and complete genomes of the *Harveyi* and *Orientalis* clades: *V. parahaemolyticus* RIMD 2210633 and *V. tubiashii* ATCC 19109, respectively, to evaluate gene conservation among the *Harveyi* and *Orientalis* clades. Genes previously predicted, were mapped in this comparison, for the identification of genomic islands (GIs) and several common characteristics of the genome, such as abnormal sequence composition or the presence of mobile genetic elements using Pfam. The gene comparison was represented by a Venn diagram (Fig. 6 inset) only on the genes that belong to a subsystem annotation in RAST.

Dot plots were generated by Gepard^56^ using default parameters with the ordered contigs produced by MAUVE for each genome.

### Genomic islands identification

Genomic islands were predicted in the draft genome of BA55 strain using IslandPick, IslandPath. DIMOB and SIGI-HMM algorithms, performed in an integrated interface from IslandViewer^57^.

### Average nucleotide identity, correlation indexes and DDH estimates

Current bioinformatics methods allow replacement of the wet-lab DNA:DNA hybridization (DDH) estimates by *in-silico* genome-to-genome comparison and transforms the distance from high-scoring segments pairs to values analogous to DDH using a generalized linear model^58^, this logistic regression model is inferred from an empirical reference dataset comprising real DDH values and pairwise genome sequences comparison. DDH *in silico* was used to obtain a better resolution of discrimination and simultaneously allow the construction of databases that permit the retrieval of any information for comparative purposes^59^.

In addition to DDH *in silico*, the metrics ANIb, ANIm and TETRA have been recently developed and are widely used as metrics to measure the distance between closely related species, generating boundaries and differentiating isolates of the same species to those of different species. The average nucleotide identity (ANI) was measured between pairs of genomes, based on the BLAST algorithm (ANIb^39^), as well as on the MUMmer aligning tool (ANIm^60^). In addition, we calculated the tetranucleotide frequencies (TETRA), a statistical method which is an alignment-free process. The values for ANIb, ANIm and TETRA for the *Vibrio* genome comparisons were calculated for all possible bacterial pairs, including all species in *Orientalis* clade (five members), one species of *Harveyi* clade (*V. parahaemolyticus*) and our BA55 strain. All comparisons were represented as heatmaps using R statistical software^35^.

**Equipment and adjustments**

**Histological Images:**

Biological Microscope OLYMPUS CX31, UIS (Universal Infinity System) optical system.

Infinity capture imaging software Lumenera Corporation, Trademark INFINITY 2.

Size: 1616 × 1216 pixels

Resolution: 96 DPI

**Gel Images:**

Equipment ENDUROtm GDS TOUCH Labnet

The Endurotm GDS Touch Aquisition Software.

Size: 768 × 526 pixels

Resolution: 96 DPI

**Organism Images:**

Ilumina Nokia 630

Size: 2592 × 1956 pixels

Resolution: 72 DPI.

The Fig. 1 is a merge of captures using photoshop CC 2015. The images were left in the same size 750 × 578 pixels. The text, arrows and contrast were modified in photoshop and the final merge was left in 300 DPI.

The Fig. 2 is a merge of captures using photoshop CC 2015. The images were left in the same size 2400 × 1644 pixels. The text, arrows and contrast were modified in photoshop. The final figure was left in 300 DPI.

## Electronic supplementary material

Supplementary Information

Table S1, Table S2, Table S3, Table S4, Table S5 and Table S6

## Data Availability

The genome sequences used in the current study are available on the NCBI Genome Database under the accession number PRJNA412371.
