# Supplementary material for: PirVP genes causing AHPND identified in a new Vibrio species (Vibrio punensis) within the commensal Orientalis clade
Source: Sci Rep. 2018 Aug 30;8:13080. doi: 10.1038/s41598-018-30903-x (PMC6117253; doi:10.1038/s41598-018-30903-x)

Pir^{VP} **genes causing AHPND identified in a new Vibrio species (*Vibrio punensis*) within the commensal *Orientalis* clade**

Leda Restrepo[a,b,c*], Bonny Bayot[a,d], Sebastián Arciniegas[e], Leandro Bajaña[a], Irma Betancourt[a], Fanny Panchana[a] and Alejandro Reyes Muñoz[b,c,f].

Figure S1. Phylogenetic reconstruction based on 16S rRNA gene, confirmed that the BA55 strain did not belong to the *Harveyi* clade. Percentage bootstrap values (1000 replicates) >85% are shown. The reference sequences were described by Sawabe et al.[5] for *Harveyi* and *Orientalis* clades. Our results show that the BA55 strain grouped together with *V. brasiliensis* which is the clade with the majority of members of the *Orientalis* clade (red lines). Nevertheless, phylogenetic reconstruction based on 16S rRNA does not have sufficient resolution (**Fig. S3**).

Figure S2. Detection of *Pir*^{VP} genes associated with AHPND in *P. vannamei*. Lane 1: Macerates from hepatopancreatic tissue from shrimp belonging to BA55 strain of the challenge assay; lane 2: Macerates from hepatopancreatic tissue from shrimp belonging to negative controls of the challenge assay; lane 3: TSB stock for the negative control used in the challenge assay; lane 4: Macerates from hepatopancreatic tissue from shrimp belonging to *V. parahaemolyticus* BA94C2 strain of the challenge assay; lane M: DL1000 DNA marker.

Table S1. *Vibrio* genomes used in this study.

Figure S3. Phylogenetic reconstruction based only on 16S rRNA. Percentage bootstrap values (one thousand bootstrap replicates). Our results show that the phylogenetic reconstruction based on 16S rRNA does not have sufficient resolution to evaluate the relationship between *Vibrio* species.

Table S2. Genomic islands (GIs) characterized for the strain *V. punensis* BA55.

Figure S4. Phylogenetic reconstruction based on concatenated FtsZ, gapA, mreB, topA, and 16S rRNA sequences to evaluate the relation between species from *Orientalis* clade. The reference sequences were as described by Dubert et al.[61] A Bayesian based

phylogeny on a concatenated multiple sequence alignment using the GTR+G+I evolutionary model is shown. Percentage bootstrap values (1000 replicates) >50% are shown. Bar, 0.02 expected nucleotide substitutions per site.

Table S3. DDH, ANIb and TETRA values.

Table S4. Primers used for amplification.

Figure S5. Phylogenetic reconstruction based only on 16S rRNA gene with all non-redundant sequences for *Vibrio* species. Percentage bootstrap values (one thousand bootstrap replicates).

Table S5. Bacterial count after challenge test. Morphological characteristics of the bacteria: Large size and convex isolated (LCI); Small size and convex isolated (SCI); Large and plain isolated (LPI).

Table S6. GenBank accession numbers of the sequences of five genes (gapA, ftsZ, mreB, topA and gyrB), used for the creation of phylogenetic trees.

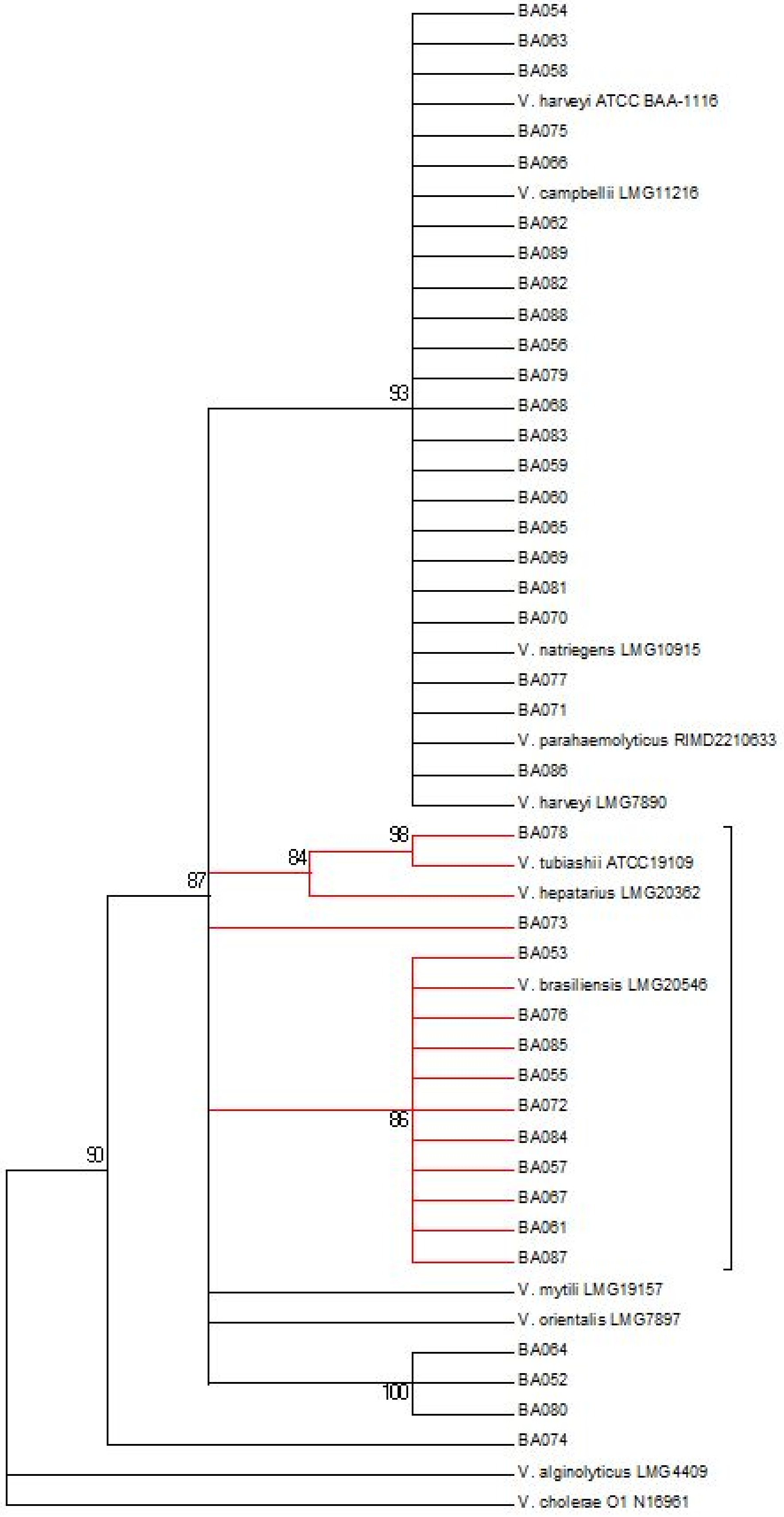

BA054
BA063
BA058
V. harveyi ATCC BAA-1116
BA075
BA066
V. campbellii LMG11216
BA062
BA089
BA082
BA088
BA056
BA079
BA068
BA083
BA059
BA060
BA065
BA069
BA081
BA070
V. natriegens LMG10915
BA077
BA071
V. parahaemolyticus RIMD2210633
BA086
V. harveyi LMG7890
BA078
V. tubiashii ATCC19109
V. hepatarius LMG20362
BA073
BA053
V. brasiliensis LMG20546
BA076
BA085
BA055
BA072
BA084
BA057
BA067
BA061
BA087
V. mytili LMG19157
V. orientalis LMG7897
BA064
BA052
BA080
BA074
V. alginolyticus LMG4409
V. cholerae O1 N16961
93
98
84
87
86
90
100

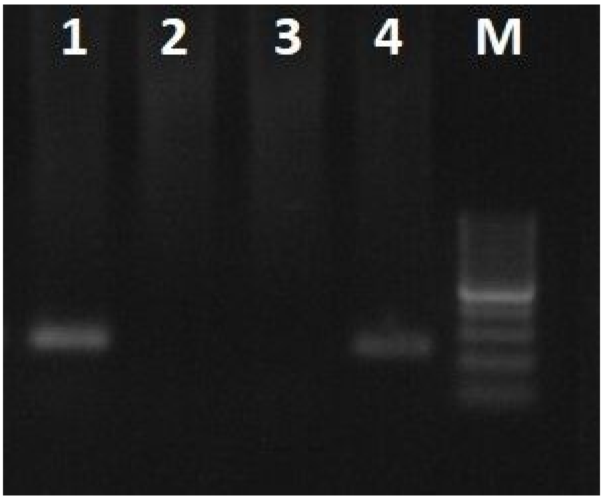
1
2
3
4
M

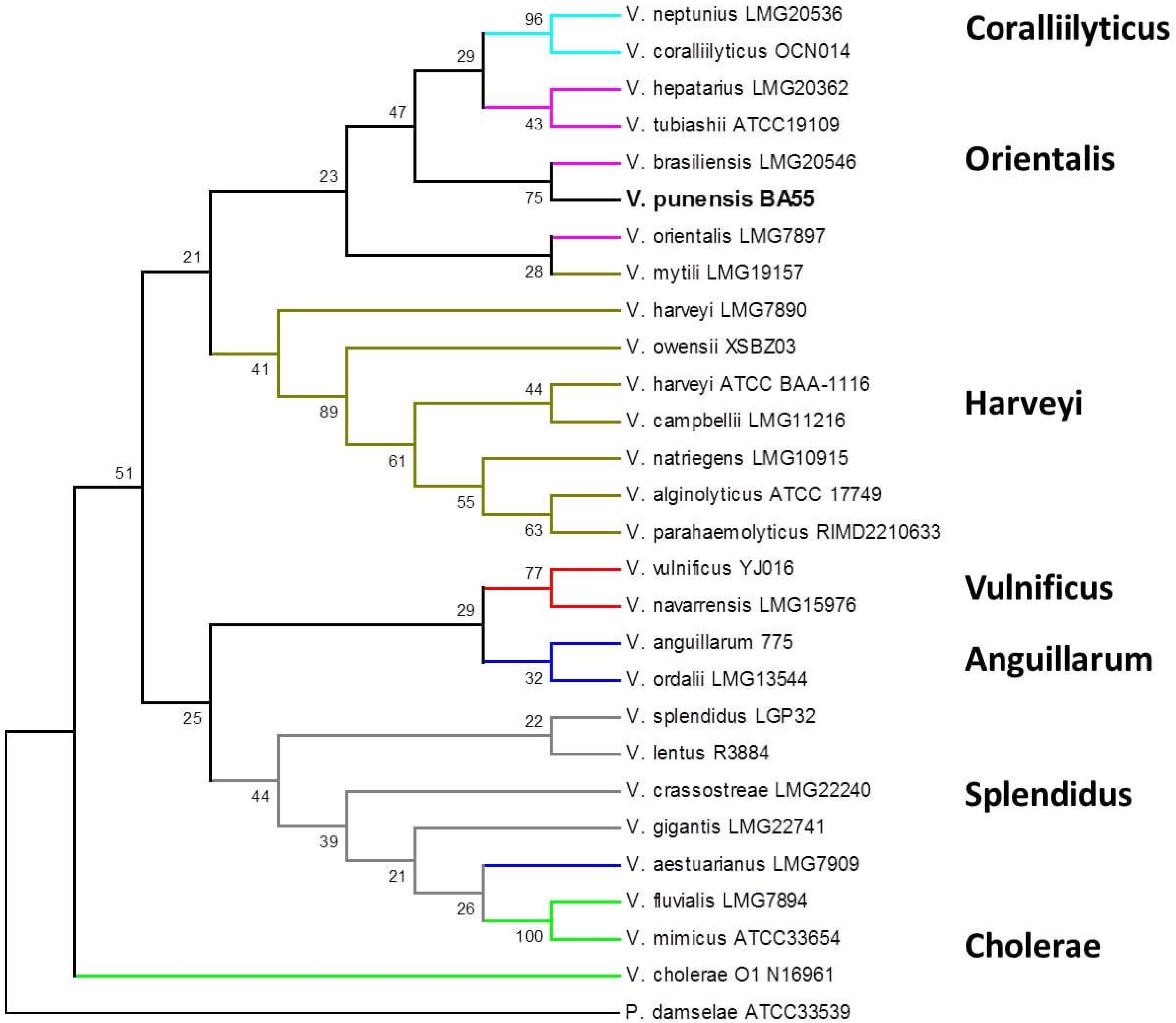

V. neptunius LMG20536
V. coralliilyticus OCN014
V. hepatarius LMG20362
V. tubiashii ATCC19109
V. brasiliensis LMG20546
V. punensis BA55
V. orientalis LMG7897
V. mytili LMG19157
V. harveyi LMG7890
V. owensii XSBZ03
V. harveyi ATCC BAA-1116
V. campbellii LMG11216
V. natriegens LMG10915
V. alginolyticus ATCC 17749
V. parahaemolyticus RIMD2210633
V. vulnificus YJ016
V. navarrensis LMG15976
V. anguillarum 775
V. ordalii LMG13544
V. splendidus LGP32
V. lentus R3884
V. crassostreae LMG22240
V. gigantis LMG22741
V. aestuarianus LMG7909
V. fluvialis LMG7894
V. mimicus ATCC33654
V. cholerae O1 N16961
P. damselae ATCC33539
Coralliilyticus
Orientalis
Harveyi
Vulnificus
Anguillarum
Splendidus
Cholerae
96
29
47
43
23
75
21
28
41
89
44
61
55
63
51
77
29
32
25
22
44
39
21
26
100

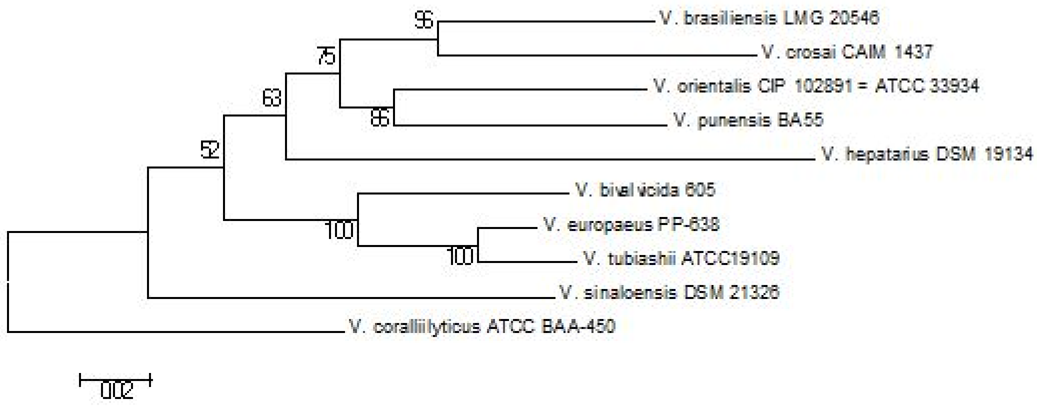
V. brasiliensis LMG 20546
V. crosai CAIM 1437
V. orientalis CIP 102891 = ATCC 33934
V. punensis BA55
V. hepatarius DSM 19134
V. bivalvicida 605
V. europaeus PP-638
V. tubiashii ATCC19109
V. sinaloensis DSM 21326
V. coralliilyticus ATCC BAA-450
96
75
86
63
52
100
100
0.02

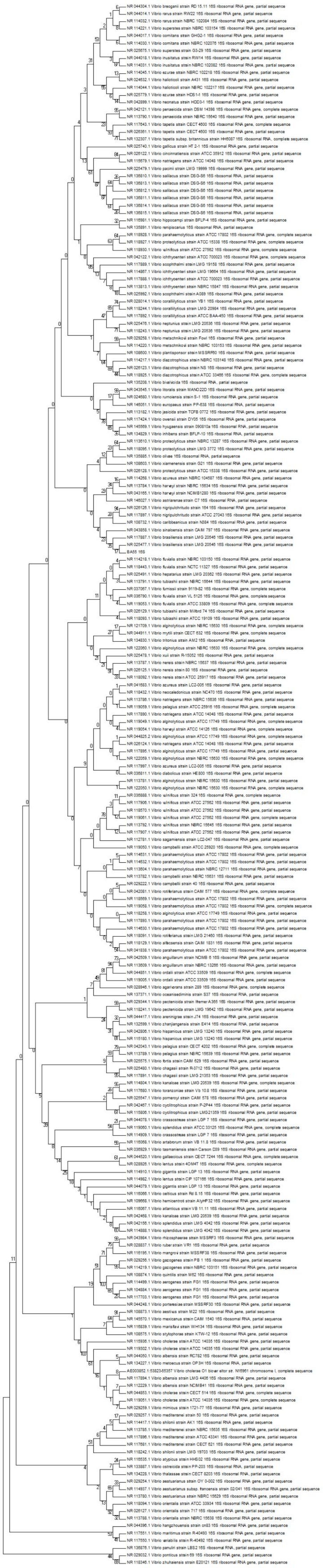

Supplement: Supplementary file 1 — Supplementary Information [file 41598_2018_30903_MOESM1_ESM.pdf]
